# Stroke-like onset of brain stem degeneration presents with unique MRI sign and heterozygous *NMNAT2* variant: a case report

**DOI:** 10.1186/s40035-016-0069-x

**Published:** 2016-12-27

**Authors:** Alexander Schulz, Franziska Wagner, Martin Ungelenk, Ingo Kurth, Christoph Redecker

**Affiliations:** 1 Hans Berger Department of Neurology, Jena University Hospital, Friedrich Schiller University Jena, Jena, 07747 Germany; 2 Present address: Department of Genetics and Program in Cellular Neuroscience, Neurodegeneration and Repair, Yale University School of Medicine, New Haven, CT 06510 USA; 3 Institute of Human Genetics, Jena University Hospital, Friedrich-Schiller-University Jena, Jena, 07743 Germany; 4 Present address: Institute of Human Genetics, Uniklinik RWTH Aachen, Aachen, 52074 Germany

**Keywords:** Brain stem atrophy, Dementia, Alexander’s disease, Whole-exome sequencing, Kissing swan sign, NMNAT2, Axon degeneration

## Abstract

**Background:**

Acute-onset neurodegenerative diseases in older patients are rare clinical cases, especially when the degeneration only affects specific regions of the nervous system. Several neurological disorders have been described in which the degeneration of brain parenchyma originates from and/or primarily affects the brain stem. Clinical diagnosis in these patients, however, is often complicated due to a poor understanding of these diseases and their underlying mechanisms.

**Case presentation:**

In this manuscript we report on a 73-year-old female who had experienced a sudden onset of complex neurological symptoms that progressively worsened over a period of 2 years. Original evaluation had suggested a MRI-negative stroke as underlying pathogenesis. The combination of patient’s medical history, clinical examination and exceptional pattern of brain stem degeneration presenting as “kissing swan sign” in MR imaging was strongly suggestive of acute onset of Alexander’s disease. This leukoencephalopathy is caused by *GFAP* (glial fibrilary acidic protein) gene mutations and may present with brain stem atrophy and stroke-like onset of symptoms in elderly individuals. However, a pathognomonic *GFAP* gene mutation could not be identified by Sanger sequencing.

**Conclusions:**

After an extended differential diagnosis and exclusion of other diseases, a definite diagnosis of the patient’s condition presently remains elusive. However, whole-exome sequencing performed from patient’s blood revealed 12 potentially disease-causative heterozygous variants, amongst which several have been associated with neurological disorders in vitro and in vivo – in particular the axon degeneration-related *NMNAT2* gene.

## Background

Neurodegenerative diseases can selectively target subpopulations of neurons in the CNS, leading to the progressive failure of defin22ed brain systems with consecutive disease-specific clinical features [1]. In various known neurological disorders, the degeneration of brain parenchyma originates from and/or primarily affects the brain stem, such as in olivopontocerebellar atrophy (OPCA), progressive supranuclear palsy (PSP), several spinocerebellar ataxia (SCA) types and various entities of leukodystrophies [2–4]. Most of these disorders have been directly or indirectly linked to specific gene mutations.

Even for long-lasting progressive neurodegenerative diseases, it has been shown that the actual cell death of neurons occurs rapidly over several hours [5]. Importantly, genetic mutations known to provoke specific neurodegenerative disorders often accelerate the very same molecular cascades that occur in late-onset types of the same diseases where those mutations cannot be found (with a classical example being Parkinson’s disease).

## Case presentation

A 73-year-old woman presented with a two-year history of progressive memory loss, urinary incontinence and walking difficulties. She had presented to an outside facility two years prior with acute onset of symptoms including a fall event. At the time of her initial presentation, she was diagnosed and reported as having had a stroke with negative findings in native CT, CT angiography and CT perfusion, as well as brain MR imaging (“MRI-negative stroke”) [6]. Clinical examination of the patient at our clinic now revealed a spastic-ataxic gait with positive pyramid signs and Romberg’s test, scanning speech, disturbed fine motor skills and dysmetria of all extremities.

Neuropsychological investigation found memory deficits suggestive of cortical dementia. Cerebrospinal fluid analysis, however, was normal with regard to both dementia and tissue destruction markers (Aβ42, total tau and phospho tau). Neither orthostatic nor autonomic dysfunction could not be detected. The possibility of a paraneoplastic syndrome was addressed by performing extensive screening for malignant tumors and known autoantibodies using imaging and serological evaluation.

**Fig. 1:**
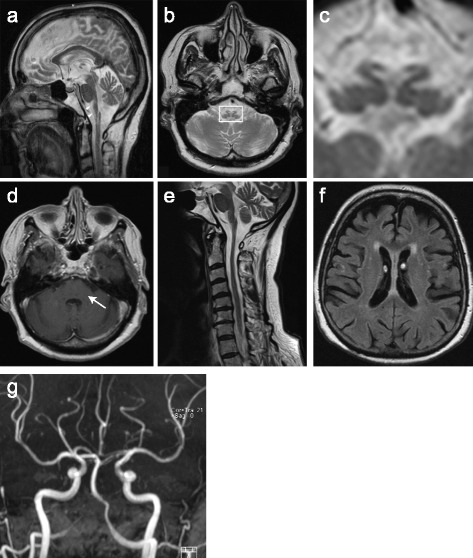
Brain stem degeneration presents in brain MRI. Sagittal (**a**) and transversal (**b**) T2-weighted brain MRI indicates gliotic neurodegeneration in the medulla oblongata with predominant loss of pyramidal tracts. Magnification of inlay (**c**) uncovers pathologic brain stem formation reminiscent of ‘kissing swans’. **d** Transversal T1-weighted sectioning of the brain stem (arrow) after Gadolinium administration. No contrast enhancement is detectable. **e** Sagittal T2-weighted cervical spine MRI shows atrophy of the upper cervical spinal cord in addition to medulla oblongata atrophy. **f** Transversal T2-weighted brain MRI indicates putative periventricular rim-sign and global brain atrophy. **g** Brain MRI-angiography reveals normal intracranial vascular status without indication of stenosis

Strikingly, brain MR imaging revealed a general atrophy of brain parenchyma with remarkable emphasis on the brain stem region (Fig. 1a and b). Here, a manifest gliotic neurodegeneration in the medulla oblongata with predominant loss of pyramidal tracts presented as “tadpole” appearance – a radiological sign exclusively described in association with Alexander’s disease [7] (Fig. 1a). Even more prominently, pattern of brain stem degeneration resembled “kissing swans” in coronal sections (Fig. 1c). Importantly, no contrast enhancement could be observed in the brain stem or any other part of the brain following Gadolinium administration (Fig. 1d), indicating blood-brain barrier integrity. Furthermore, a marked atrophy of the upper cervical spinal cord was detected (Fig. 1e).

DaTscan/IBZM SPECT and FDG-PET results were neither indicative of idiopathic nor atypical Parkinson’s disease (not shown).

The combination of patient history and characteristic brain stem degeneration was strongly reminiscent of case reports of acute-onset adult Alexander’s disease (AOAD) [8]. Alexander’s disease is a rare form of leukodystrophy, characterized by white matter degeneration, mainly caused by gain-of-function mutations of the glial fibrillary acidic protein (GFAP) in astrocytes [9].

Although most cases were reported to occur sporadically, autosomal dominant transmissions have been described [10]. The family history of this patient was devoid of similar cases, with the important limitation that the patient had been adopted along with her siblings and has no children herself. However, personal reports as well as brain MR imaging findings of the patient’s sister, niece and grandniece gave no indication of a comparable disease in the patient’s family.

In order to genetically examine the suspected diagnosis of Alexander’s disease, we performed Sanger sequencing of all coding exons and adjacent intronic regions of the *GFAP* gene from the patient’s blood. Strikingly, we could not identify any mutations in the *GFAP* gene. Specific genetic testing for the most frequent spinocerebellar ataxia (SCA) types [11] was also negative.

We therefore performed whole-exome sequencing from patient’s blood, which provides an unbiased analysis of all protein-coding sequences in the human genome. Stringent filtering of whole-exome sequencing data for rare genetic variants (using dbSNP, 1000 genomes project, the Exome Variant Server and ExAC browser) as well as for disease-causative variants (using SIFT, Polyphen 2 and Mutation taster 2) resulted in a list of 12 potentially disease-causative heterozygous variants (Table 1).

**Table 1:** List of potentially disease-causative, heterozygous variants based on whole-exome sequencing indicating gene (Gene), reference sequence (RefSeq), mutation within the gene sequence (MutCDNA), position of the mutation within the genome according to hg38 (Position), mutation within the protein sequence (MutProt), type of mutation (Type), chromosome location (Chr), allele frequency (AL), number of affected exon (MutPos) and consequence of mutation (Consequence)

Gene	RefSeq	MutCDNA	Position	MutProt	Type	Chr	AF	MutPos	Consequence
HIST1H2AG	NM_021064.4	c.1A > G	27100851	p.M1V	SNP	6	het	CDS.1	Start codon lost
KAT5	NM_001206833.1	c.164A > G	65479902	p.N55S	SNP	11	het	CDS.1	Non-synonymous coding
LGR5	NM_003667.2	c.2611_2613del(T)3ins(T)2	71978401	p.F871Lfs*22	INDEL	12	het	CDS.18	Frameshift
LIMCH1	NM_001112719.1	c.20_21insG	41615112	p.K8Qfs*4	INS	4	het	CDS.1	Frameshift
LPPR3	NM_024888.1	c.1579G > A	813232	p.E527K	SNP	19	het	CDS.7	Frameshift
MFF	NM_020194.4	c.696delG	228212044	p.Q232Hfs*46	DEL	2	het	CDS.8	Frameshift
MLNR	NM_001507.1	c.762_764delCTA	49795235	p.Y255del	DEL	13	het	CDS.1	Deletion
MYH7B	NM_020884.3	c.3425_3428delGGGTins (GGGT)2	33584918	p.E1144Gfs*130	INDEL	20	het	CDS.29	Frameshift
NMNAT2	NM_170706.3	c.482 T > C	183253877	p.V161A	SNP	1	het	CDS.6	Non-synonymous coding
PRDM16	NM_199454.2	c.1093G > C	3322119	p.A365P	SNP	1	het	CDS.8	Non-synonymous coding
STOX1	NM_152709.4	c.2341G > T	70645893	p.E781*	SNP	10	het	CDS.3	Stop codon gained
TRIM41	NM_201627.2	c.424_447delGAGGAGGACCTGAGGGGGGAGGAT	180651423	p.E142_D149del	DEL	5	het	CDS.1	Deletion

The most notable of these candidate genes was *NMNAT2*, which encodes for one isoform of the nicotinamide mononucleotide adenylyltransferases. Its neuroprotective role in numerous preclinical models of neurodegeneration is well established. Experimental deletion of *NMNAT2* function increases the vulnerability of CNS neurons and accelerates Wallerian degeneration [12]. Furthermore, *NMNAT2* transcript levels have been shown to negatively correlate with neurodegeneration in a large study of 541 human individuals [13]. Importantly, deletion of a single *NMNAT2* allele, analogous to the heterozygous variant found in the presented patient, was sufficient to induce neurite degeneration in primary neuronal cultures [14].

Another candidate gene, *STOX1* has been associated with phosphorylation of tau proteins in late-onset Alzheimer’s disease [15]. *KAT5*, in addition, acts as a nuclear hormone receptor coactivator and has been linked to spinocerebellar ataxia type 1 [16].

## Discussion

Acute onset of chronic neurodegenerative diseases such as dementia [17] or movement disorders [18] in elderly individuals can occur as a result of various pathological conditions. However, the mechanisms determining an acute disease course as opposed to a slowly-progressive one for a specific disease are incompletely understood. For these and other reasons, both the definite diagnosis and the underlying cause of the presented patient’s condition are yet unknown. The diagnosis of adult-onset Alexander’s disease (AOAD) as a rare leukoencephalopathy disorder predominantly affecting the brain stem and having stroke-like onset of symptoms [8] has to be excluded in this patient’s case, due to the apparent absence of a detectable *GFAP* gene mutation; nevertheless, the unique combination of clinical presentation, patient history and severe degeneration of the medulla oblongata are consistent with other reports on the disease.

It therefore needs to be resolved in future studies whether Alexander’s disease can be caused by mutations in genes other than *GFAP*, or whether mutations in other genes can lead to comparable but independent disease entities. In fact, phenotypically comparable presentations of adult onset ataxia, dementia, and typical MRI findings (tadpole sign) have been described in *GFAP* mutation-negative patients [19].

Remarkably, the original diagnosis in this case (2 years prior to the current examination) was MR-negative stroke, thereby addressing the sudden onset of symptoms. Yet, in the current follow-up evaluation, no vascular pathologies (Fig. 1f and g) or cardiovascular risk factors (diabetes mellitus, cardiac arrhythmia, smoking, hyperlipidemia) except for mild hypertension could be determined. Furthermore, several of the initial symptoms (spasticity and memory loss in particular) were reported to be steadily progressive by the patient.

Other possible differential diagnoses as underlying cause for the patient’s condition (multiple system atrophy, progressive supranuclear palsy, spinocerebellar ataxia or paraneoplastic/immunological syndrome etc.) appear unlikely at this time, based on the existing information.

## Conclusions

Despite an extended differential diagnosis and exclusion of other possible diseases, a definite diagnosis of the presented case remains elusive. The diagnosis of adult-onset Alexander’s disease predominantly affecting the brain stem with stroke-like onset of symptoms has to be excluded, because of the apparent absence of a detectable *GFAP* gene mutation. Yet, stringent filtering of whole-exome sequencing data reveals 12 potentially disease-causative heterozygous variants, which may impact the patient’s condition. Additional cases or disease-mimicking animal models with targeted gene knockout or over-expression of candidate genes will be necessary to clarify a disease-causative potential of the candidates shown in Table 1.

However, the identification of several genes with experimentally proven connection to neurodegenerative diseases, in particular the axon degeneration-related gene *NMNAT2*, as well as the uniqueness of this case suggest a genetic cause for the patient’s debilitating and progressive condition.

## Abbreviations

AOAD: Adult-onset Alexander’s disease
CNS: Central nervous system
DaTscan: Dopamine transporter scan
Del: Deletion
FDG: Fluorodeoxyglucose
GFAP: Glial fibrilary acidic protein
IBZM: Iodobenzamide
Indel: Insertion/Deletion
MRI: Magnetic resonance imaging
NMNAT2: Nicotinamide nucleotide adenylyltransferase 2
OPCA: Olivopontocerebellar atrophy
PET: Positron emission tomography
PSP: Progressive supranuclear palsy
SCA: Spinocerebellar ataxia
SNP: Single nucleotide polymorphism
SPECT: Single-photon emission computed tomography
STIR: Short tau inversion recovery.
